# A versatile, compartmentalised gut-on-a-chip system for pharmacological and toxicological analyses

**DOI:** 10.1038/s41598-021-84187-9

**Published:** 2021-03-01

**Authors:** Pim de Haan, Milou J. C. Santbergen, Meike van der Zande, Hans Bouwmeester, Michel W. F. Nielen, Elisabeth Verpoorte

**Affiliations:** 1 Pharmaceutical Analysis, Groningen Research Institute of Pharmacy, University of Groningen, P.O. Box 196, XB20, 9700 AD Groningen, The Netherlands; 2 TI-COAST, Science Park 904, 1098 XH Amsterdam, The Netherlands; 3 Laboratory of Organic Chemistry, Wageningen University, Stippeneng 4, 6708 WE Wageningen, The Netherlands; 4 Wageningen Food Safety Research, Wageningen University & Research, P.O. Box 230, 6700 AE Wageningen, The Netherlands; 5 Division of Toxicology, Wageningen University, Stippeneng 4, 6708 WE Wageningen, The Netherlands

**Keywords:** Lab-on-a-chip, Mass spectrometry, Gastrointestinal models, Toxicology, Pharmacokinetics, Lab-on-a-chip, Microfluidics, Mass spectrometry

## Abstract

A novel, integrated, in vitro gastrointestinal (GI) system is presented to study oral bioavailability parameters of small molecules. Three compartments were combined into one hyphenated, flow-through set-up. In the first compartment, a compound was exposed dynamically to enzymatic digestion in three consecutive microreactors, mimicking the processes of the mouth, stomach, and intestine. The resulting solution (chyme) continued to the second compartment, a flow-through barrier model of the intestinal epithelium allowing absorption of the compound and metabolites thereof. The composition of the effluents from the barrier model were analysed either offline by electrospray-ionisation-mass spectrometry (ESI–MS), or online in the final compartment using chip-based ESI–MS. Two model drugs, omeprazole and verapamil, were used to test the integrated model. Omeprazole was shown to be broken down upon treatment with gastric acid, but reached the cell barrier unharmed when introduced to the system in a manner emulating an enteric-coated formulation. In contrast, verapamil was unaffected by digestion. Finally, a reduced uptake of verapamil was observed when verapamil was introduced to the system dissolved in apple juice, a simple food matrix. It is envisaged that this integrated, compartmentalised GI system has potential for enabling future research in the fields of pharmacology, toxicology, and nutrition.

## Introduction

A main entry route of compounds required by the body for its well-being is via the mouth, with these compounds contained in foods and medicines. A material that is ingested undergoes a series of processes which result in the liberation of the compounds of interest from the material matrix and their entry into the circulatory system. Once in the circulatory system, they find their way to other locations in the body where they will be most effective. The fraction of orally ingested compounds that makes it into the circulatory system is defined as the oral bioavailability^1^. The oral bioavailability of ingested compounds is thus of nutritional, pharmacological and toxicological interest. It is a crucial factor, for example, for drug dosing in pharmacotherapy, in the occurrence of oral intoxications, and in nutrition studies. Oral bioavailability is determined by three processes, namely bioaccessibility, intestinal absorption and metabolism by enzymes in gut or liver cells. A chemical is considered to be bioaccessible if a fraction of it has been released from the ingested matrix (e.g. food, drug, etc.) in a form that can be absorbed by the intestinal wall^2^. Parameters in the gastrointestinal (GI) tract, such as pH, enzymatic content, and residence time, are of great influence on the bioaccessibility of a chemical, and there are several in vitro digestion models available to study this. These models mimic the chemical and enzymatic reactions that take place in the mouth, stomach and small intestine^3,4^. In the mouth phase, the main process involved in digestion can be ascribed to the enzyme amylase, which digests starch^5^. In the stomach phase, a low pH causes denaturation of proteins (as well as damage to other acid-labile chemical bonds), which are then hydrolysed into smaller peptides by the enzyme, pepsin^6^. In the small intestine, the pH is neutralised by the addition of bicarbonate. Enzymes from the pancreas, including proteases and lipases, are also introduced^7^, as is bile from the gallbladder to emulsify fats^8^. These latter additions complete the formation of a digestive mixture known as chyme. It is this mixture that enters the intestine, and from which compounds are absorbed through the intestinal wall.

The next process involved in determining oral bioavailability is absorption by the intestinal wall. First-pass metabolism by the intestine and liver is sometimes also included in the definition of oral bioavailability^1^. However, in this study on bioavailability we have focused only on absorption of a compound through the intestinal wall, which may occur via mechanisms classified as either active or passive. Currently, static in vitro cell culture models are used to predict oral bioavailability^9^. Static in vitro cell culture models of the intestine, based on immortalized human cell lines such as Caco-2 cells, have been used in the past to provide data for human intestinal uptake^10,11^. Caco-2 cell layers have been found to provide good predictability for absorption of small lipophilic drugs that are commonly absorbed through transcellular diffusion when cultured in static transwell systems. Transwell systems are based on porous membrane inserts immersed in wells to create apical (above the membrane) and basolateral (below the membrane) volumes, with cell layers cultured on the porous membrane in the apical compartment. Test compounds are transported passively by diffusion to the membrane. Transwell models thus do not capture dynamic features, such as peristaltic motion and flow-induced shear stress present in the intestine.

The rise of microfluidic technology and organ-on-a-chip devices provides an opportunity to miniaturize the systems used for bioavailability studies. One example is the recent development of a three-stage, flow-based digestion-on-a-chip model for determination of bioaccessibility, where chemical and enzymatic breakdown in the mouth, stomach and intestinal phase is recapitulated^12^. Each digestive chamber is individually addressable so that conditions can be tuned with respect to pH, enzyme activity, and other parameters. Moreover, a range of different dynamic cell culture systems which mimic the intestinal epithelial barrier has been developed for compound permeability studies^13–19^. These systems share a common design consisting of two chambers separated by a porous membrane containing cells of human intestinal origin. The cells used are either from cell lines, or less frequently, primary human cells or cells derived from human stem cells. The cells inside the devices are subjected to flow (hence the designation “dynamic”), resulting in a better representation of the in vivo intestinal microenvironment by inducing shear stress on the cells^18,20^. Absorption of chemical compounds found in drugs and nutrients has been investigated in some of these dynamic cell culture systems by integrating them with analytical detection platforms allowing for automated real-time measurements and identification of (un)known metabolites^17,21–25^. Two examples of systems that approach what we show in this paper with respect to digestion coupled to absorption are known. In one case, there were enzymes and bile salts in the digestion medium being flushed through the absorption module^24^. Though these examples represent a high level of compartmentalisation, analysis of processes running within them were not done in real-time or online^24,25^.

In this study, we have taken the development of these in vitro systems one step further to open up a route to a more complete analytical approach to determine oral bioavailability, before possible first-pass metabolism by the liver^1^. We propose that an ideal, automated platform to study oral bioavailability should consist of a digestion-on-a-chip coupled to an in vitro, flow-through, intestinal epithelial model which in turn is coupled to mass spectrometric (MS) detection. This is highly challenging for a number of reasons: (1) The relatively high levels of digestive enzymes and bile salts present in the undiluted chyme coming from the digestion-chip are toxic to the intestinal epithelial cells in the absorption compartment. (2) Volumetric flow rates in the different compartments should be matched to ensure that the resulting shear stress is within the physiological range. (3) The physiological concentrations of proteins and salts in the chyme interfere with MS analysis, and must be removed by sample pretreatment to minimize background chemical noise^26^. As a first demonstration of our system, we have chosen to determine the bioavailability parameters of two small-molecule drugs, omeprazole and verapamil. These two drug molecules were chosen to benchmark our system because of their well-known behaviour in the GI tract. Omeprazole is normally administered as an enteric-coated formulation, as the molecule is sensitive to acidic degradation in the stomach^27,28^. The fate of omeprazole was studied using either full digestion (i.e., mouth, stomach, and intestine), or a simplified, intestine-only digestion. Verapamil, on the other hand, is unaffected by the digestive processes; this drug was studied in the presence of a simple food matrix to study possible effects thereof.

## Materials and methods

### Chemicals

Verapamil hydrochloride, omeprazole, penicillin–streptomycin, formic acid, lucifer yellow, 4-(2-hydroxyethyl)-1-piperazineethanesulfonic acid (HEPES), sodium bicarbonate, Triton-X100 and Hank’s balanced salt solution (HBSS), with and without phenol red, were all purchased from Sigma-Aldrich/Merck (Zwijndrecht, the Netherlands). Dulbecco’s Modified Eagle Medium (DMEM) with 4.5 g/L glucose and L-glutamine with and without phenol red, bovine serum albumin (BSA) and heat-inactivated fetal bovine serum (FBS) were obtained from Gibco (Bleiswijk, the Netherlands). Rabbit polyclonal antibody ZO-1/TJP1 conjugated to Alexa fluor 594, Prolong Diamond Antifade Mountant, dimethyl sulfoxide (DMSO), phosphate-buffered saline (PBS) and non-essential amino acids (NEAA) were bought from Thermo Fisher Scientific (Landsmeer, the Netherlands)^23^. All chemicals for digestive juices, including enzymes, came from Sigma-Aldrich/Merck, except sodium dihydrogen phosphate monohydrate and hydrochloric acid (Acros, Geel, Belgium), and potassium chloride and sodium chloride (Duchefa, Haarlem, the Netherlands). Acetonitrile was purchased from Actu-All Chemicals (Oss, the Netherlands), and Ultrahigh Pressure Liquid Chromatography-Mass Spectrometry (UPLC-MS) grade water from Biosolve (Valkenswaard, the Netherlands). Paraformaldehyde was obtained from VWR (Amsterdam, the Netherlands), polydimethylsiloxane (PDMS) from Dow Corning (Sylgard, Midland, Michigan, USA), and WST-1 reagent from Roche Diagnostics GmbH (Mannheim, Germany). Water was prepared fresh daily using a Milli-Q Reference Water Purification System from Millipore (Burlington, Massachusetts, USA).

### Cell culture

The human colorectal adenocarcinoma cell line, Caco-2, was obtained from the American Type Culture Collection (ATCC, Manassas, Virginia, USA) and co-cultured with the human colon adenocarcinoma mucus secreting cell line HT29-MTX-E12 obtained from the European Collection of Authenticated Cell Cultures (ECACC, Salisbury, UK). Cells were used at passage numbers 29–40 (Caco-2) and 52–70 (HT29-MTX-E12). Cell lines were cultured in separate, 75 cm^2^, cell culture flasks (Corning Inc., Corning, New York, USA) in cell culture medium (DMEM containing 10% FBS, penicillin–streptomycin (100 U/mL and 100 µg/mL) and 1% NEAA). Cells were maintained in a humidified 5% CO_2_ atmosphere at 37 °C and subcultured every 2 to 3 days. The cells were seeded at a density of 40,000 cells/cm^2^ on a polycarbonate 24-well transwell insert (0.4 µm pore size, 0.6 cm^2^ surface area, Millipore) in cell culture medium. Caco-2 and HT29-MTX-E12 cells were seeded on the apical side of the insert at a 3:1 ratio; cell culture medium was replaced every other day. For permeability experiments, transwell inserts were placed into the QV600 system from Kirkstall (Rotherham, UK), henceforth referred to as a flow-through transwell, at day 20 of culture. The apical and basolateral compartments of the flow-through transwell each had internal volumes of 2 mL. Cell culture medium containing 25 mM HEPES was introduced into the apical compartment (200 µL/min, maximum shear stress 6 × 10^–3^ dyne/cm^2^^29^) and basolateral compartment (100 µL/min) of the flow-through transwell system, using a separate syringe pump (New Era Pump Systems, Farmingdale, New York, USA) for each compartment. Medium was maintained at 37 °C and perfusion continued for 24 h, as described by Giusti et al.^29^. After 24 h, the apical and basolateral syringes were both replaced by syringes containing HBSS with 25 mM HEPES and 0.35 g/L NaHCO_3_ added. To assure a biologically relevant environment, syringe heaters (New Era Pump Systems) were used to heat the medium and keep the cells at 37 °C without the need for an additional incubator.

### Cell viability

Possible cytotoxic effects of omeprazole and chyme were evaluated using the WST-1 cell viability assay. First, Caco-2 and HT29-MTX-E12 cells (ratio 3:1) were seeded in flat bottom 96-well plates (Greiner Bio-One, Alphen aan den Rijn, the Netherlands) at a concentration of 1 × 10^5^ cells/mL in cell culture medium (100 µL/well). Plates were incubated at 37 °C under 5% CO_2_ for 24 h. Cell culture medium was removed, and the cells were subsequently exposed for 24 h to 100 µL/well volumes of serial dilutions of omeprazole (0–50 µg/mL) or chyme (0–100%) in cell culture medium at 37 °C. Then, the exposure media containing the compounds was discarded and the cells were washed with pre-warmed HBSS. Subsequently, WST-1 reagent (in cell culture medium without phenol red, as this interferes with absorbance measurements) was added to the cells (1:10, 100 µL/well). After 1.5 h of incubation at 37 °C, the absorbance of each well was measured at 440 nm using a Synergy HT Multi-Mode microplate reader (Bio-Tek, Winooski, Vermont, USA). The viability of the cells for each concentration of chyme was expressed as a percentage of the negative control consisting of only cell culture medium. For omeprazole, the negative control consisted of cell culture medium with 0.5% DMSO added to match the concentration of DMSO in the samples. Triton-X100 (0.25%, v/v) was used as a positive control and decreased the cell viability to 0.0 ± 0.4%.

### Evaluation of cell barrier integrity

Barrier integrity was evaluated after cells had been cultured on a transwell membrane for 21 days and subsequently stained for tight junction protein, ZO-1, as described before^23^. Just before staining, the cells were washed with PBS and fixed with 4% paraformaldehyde (w/v) for 15 min, permeabilized with 0.25% Triton-X100 (v/v) and blocked with 1% BSA (w/v). The cells were then incubated with 100 µL of solution containing 10 µg/mL of the conjugated antibody ZO-1/TJP1-Alexa Fluor 594 for 45 min. Between steps, cells were washed with PBS three times. Cells were mounted in a 120-µm-thick spacer (Sigma-Aldrich) on a microscope slide (Thermo Scientific) with ProLong Diamond Antifade Mountant. Slides were then examined using a confocal microscope (LSM 510-META, Zeiss, Jena, Germany), with samples excited with a 543 nm laser at a magnification of 40 X. Cell layer integrity was also evaluated using the transport marker, lucifer yellow. Following drug permeability experiments, the cells were incubated with lucifer yellow at an apical concentration of 500 µg/mL in HBSS for 30 min. HBSS was collected from the apical and basolateral side at t = 0 and t = 30 min and analysed for fluorescence at 458/530 nm (excitation/emission) using a microplate reader. Cell layers that transported more than 5% of lucifer yellow to the basolateral compartment were considered leaky and discarded.

### Artificial digestive juices

Artificial saliva, stomach juice, duodenal juice and bile were prepared as described by de Haan et al.^12^; a detailed composition of the juices can be found in Table S1 of the supplementary information (SI). We refer the reader to de Haan et al.^12^ for a more detailed explanation about the choices made underlying this model, with respect to the amounts of the different physiologically relevant components included in the juices. In short, all chemicals except the enzymes were dissolved in ultrapure water and the pH was evaluated using a pH meter (Metrohm 713, Barendrecht, the Netherlands) and adjusted as necessary using HCl or NaOH. Only after setting the right pH (leading to local pH values of 7.0, 3.0 and 7.0 in the mouth, stomach, and intestine compartments, respectively) were the enzymes added to the juices, this to prevent inactivation or denaturation of enzymes if added to a solution with an aberrant pH.

### Compartmentalised system design and operation

**Figure 1:**
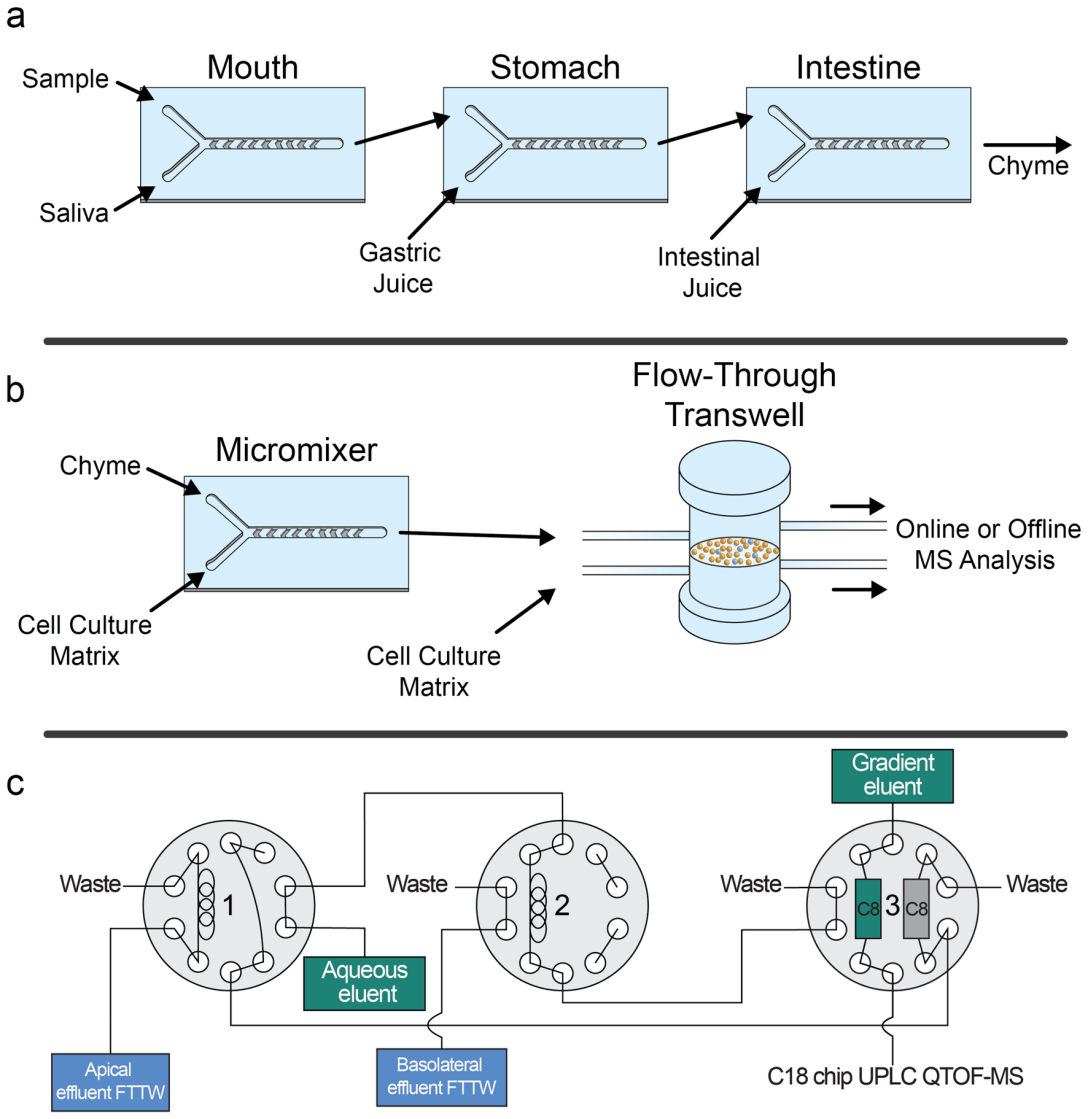
Schematic overview of the different components making up the three compartments used throughout our experiments. (**a**) Compartment 1—Digestion-on-a-chip: Three chaotic micromixers representing the mouth, stomach and intestinal phase of digestion are coupled to one another using PTFE tubing. Conditions are individually controlled by addition of appropriate artificial juices to each micromixer. (**b**) Compartment 2—Absorption: A fourth micromixer is used to mix the chyme from the digestion-on-a-chip with cell culture matrix needed for the cells, in order to dilute the chyme before exposing cells to it. This solution was introduced to the apical side of the flow-through transwell (FTTW) system containing a co-culture of Caco-2 and HT29-MTX-E12 cells. For offline analysis, a fraction collector was used to continuously obtain samples from both the apical and basolateral chambers. For online analysis, the flow-through transwell is coupled to Compartment 3 (**c**). (**c**) Compartment 3—On-line analysis with automated sample clean-up: Sample is automatically collected from the apical and basolateral chambers in the first and second switching valves, respectively. In the third switching valve, two nanotraps were integrated to retain the analyte of interest and wash away unwanted sugars and salts. Subsequently, the analyte of interest was eluted to a microfluidic C18 chip-based column and analysed by QTOF-MS. The state of the valves in this figure indicates the initial configuration at the start of the experiment, when apical effluent is being collected in the sample loop of Valve 1, while the sample loop in Valve 2 is being flushed with aqueous eluent.

Fabrication of the digestion-on-a-chip system (Compartment 1), shown in Fig. 1a, has been previously described^12^. In short, identical micromixer devices for the three phases of digestion were fabricated by micromolding PDMS on molds made by photolithography in SU-8 photoresist layers deposited on 0.7-mm-thick, polished glass substrates. Mixing channels were 300 µm wide and 51.5 mm long, and contained 16 sequential arrays of 12 herringbone-shaped grooves each embedded in the bottom of the channel structure. Channels were 60 µm deep, and 50 µm deeper in the groove regions. Grooves were 110 µm wide and spaced 60 µm apart. The total internal volume of each micromixer was 1.48 μL including inlet channels leading to the groove arrays. The grooves perturb the profile of side-by-side laminar flows entering the mixing channel to generate ‘chaotic’ flow patterns that result in larger contact areas between solutions. In this way, diffusion distances are shortened substantially and diffusional mixing times dramatically reduced. The different digestive compartments were connected to each other via polytetrafluoroethylene (PTFE) tubing (0.8/1.6 mm inner/outer diameter, Polyfluor Plastics, Breda, the Netherlands) (Fig. 1a). Flow for the digestion-on-a-chip was regulated by a pressure-driven flow control system^30^. Pressurised air was passed through a microfilter (PTFE, 0.45 µm pore size, Boom B.V., Meppel, the Netherlands) and distributed to the four glass bottles into which 15 mL tubes (Greiner Bio-One, Frickenhausen, Germany) containing the digestive juices and the sample had been placed. Digestive juices in each of the containers were kept at a constant pressure of 500 mbar. PTFE tubing was used to connect the liquid-containing glass bottles to Coriolis-based mass flow controllers (ML120 and BL100, Bronkhorst High-Tech, Ruurlo, The Netherlands), using blunt Fine-Ject 21G needles (HenkeSassWolf, Tuttlingen, Germany) fitted directly inside the open end of the tubing. The micro-Coriolis-based mass flow sensors were used to regulate the flow of juices and samples with a far greater stability and accuracy than would be possible with syringe pumps and flow sensors based on other measurement principles^30^. A Bronkhorst software package was used to change flow controller settings and to take measurements of mass flow and density. In Compartment 2, a fourth micromixer (Fig. 1b) was incorporated to dilute the chyme from the digestion-on-a-chip with the exposure medium (HBSS) required for permeability experiments, to prevent any cytotoxic effect of chyme on the cells. The effluent from this last micromixer was connected to the apical side of the flow-through transwell (Fig. 1b). Subsequently, the flow-through transwell was coupled to a fraction collector, collecting one sample every minute in a 96-well plate. Alternatively, the flow-through transwell was connected to Compartment 3, which consisted of a series of three switching valves connected to a microfluidic chip-based UPLC-QTOF-MS (Fig. 1c) as described by Santbergen et al.^23^ In this latter option, apical and basolateral effluents from the flow-through transwell were alternately loaded in 5 µL stainless steel sample loops mounted on the first and second switching valve (Fig. 1c). Each sample loop was loaded for 15 min. After sample collection, the content of each sample loop was depleted of proteins and bile salts by flushing for 4 min through an Optimize Technologies (Oregon City, Oregon, USA) C8 nanotrap column (180 µm × 5 mm, 2.7 µm) using an aqueous solvent (H_2_O with 1% acetonitrile) at a flow rate of 20 µL/min. Following the clean-up, the trap column was eluted with a microflow gradient at 3 µL/min towards a microfluidic iKey chip BEH C18 analytical column for UPLC-QTOF-MS analysis (see below for more details on MS analysis).

### Offline and online UPLC-QTOF-MS analysis

#### Offline analysis of omeprazole

For the static cell permeability experiments in transwells, 5 µg/mL omeprazole was suspended in HBSS (without phenol red) containing 25 mM HEPES and 0.35 g/L NaHCO_3_ as the donor solution. At day 21 of culture, the donor solution was directly applied to the apical side of the cells (400 µL/insert). The basolateral side was filled with 600 µL of HBSS per insert. Samples were taken (100 µL) from the basolateral side at 15, 30, 45, 60, 120 and 180 min. The sampled medium was replenished after each sample acquisition with 100 µL of fresh medium. At t = 0 and t = 180 min, apical samples were taken.

For dynamic experiments in the flow-through device, including the complete integrated modular GI tract (Fig. 1a,b), omeprazole was introduced into the digestion-on-a-chip at a concentration of 1 mg/mL in DMSO (1 µL/min). When intestinal digestion only was desired, omeprazole was dissolved in the combined digestive juices from the mouth, stomach and intestine at a concentration of 40 µg/mL before introduction to the micromixer in which chyme was diluted with HBSS. In both cases, the final omeprazole concentration on the apical side of the flow-through transwell was 5 µg/mL. The apical and basolateral effluent flows of the flow-through transwell were directed to a Gilson 234 autosampler (Villiers-le-Bel, France), which was used as a fraction collector in this case to collect samples every minute in a 96-well plate. All samples were analysed undiluted by UPLC-QTOF-MS, using the procedure that is described next. One 2-position/10-port Ultralife switching valve (IDEX Health & Science, Oak Harbor, Washington, USA) with 1/16″ fittings was used to incorporate online sample preparation with the microfluidic chip UPLC-QTOF-MS. A nano Acquity autosampler (Waters) set at 10 °C and with a 2 µL injector was used. The sample loop was flushed for 4 min with mobile phase A (water with 1% acetonitrile and 0.1% formic acid) at a flow rate of 3 µL/min towards a C8 nanotrap column (Optimize Technologies) (180 µm I.D. × 5 mm, 2.7 µm particles). Following the clean-up, the nanotrap column was eluted towards a microfluidic chip-based iKey BEH C18 analytical column (150 µm I.D. × 50 mm, particle size 1.7 µm) (Waters) by switching the valve. The 3 µL/min microflow gradient elution consisted of mobile phase A (*cf*. above) and mobile phase B consisting of acetonitrile with 1% water and 0.1% formic acid. The gradient started at 0% B, and after 1 min was increased to 50% B in 0.1 min. This composition was maintained for 3.9 min, and then increased to 90% in 0.1 min, to be kept constant for 3.9 min. The composition was returned to 0% in 0.1 min and an equilibration time of 3.9 min was allowed prior to the next injection. MS detection was performed with a Waters Xevo QTOF MS equipped with an iKey nano electrospray ionisation source operated in the positive ion mode, with a capillary voltage of 3.9 kV, desolvation temperature of 350 °C, gas flow rate 400 L/h, source temperature of 80 °C and cone gas flow rate of 10 L/h. Data were acquired and processed using MassLynx v4.1 (Waters) software.

#### Online analysis of verapamil

Verapamil was introduced into the compartmentalised GI-tract, total-analysis system at a concentration of 1 mg/mL (Fig. 1a) in either ultrapure water or apple-juice sample matrices (1 µL/min). The final concentration of verapamil on the apical side of the flow-through transwell was 5 µg/mL. The process of automated sample clean-up and trapping was described above in the section “Compartmentalised system design and operation”. In the case of verapamil analysis, the C8 nanotrap column was eluted towards a microfluidic chip-based iKey BEH C18 analytical column using the following gradient. The 3 µL/min microflow gradient was based on a published method^23^ and consisted of mobile phase A (water with 1% acetonitrile) and mobile phase B (acetonitrile with 1% water), both containing 0.1% formic acid. The gradient started at 10% B and, after 4 min, was linearly increased to 100% B in 4 min. This composition was kept constant for 3 min, and then reverted to 10% B in 0.1 min. An equilibration time of 3.9 min was allowed prior to the next injection. MS detection was performed with a Waters Xevo QTOF mass spectrometer with the same settings as for the offline analysis of omeprazole. Data were collected using MassLynx, yielding a separate data file for each trap-column analysis.

### Permeability calculations

The apparent permeability coefficient (P_app_, cm/s) was calculated as described by Yeon and Park^31^, according to the following equation:

$${P}_{app}=\frac{dQ}{dt}\frac{1}{A {C}_{0}}$$

In this equation, dQ/dt is the transport rate into the basolateral compartment (µmol/s), A is the surface area of the cell layer (0.6 cm^2^) and C_0_ is the initial concentration of the compounds in the apical compartment (µmol/cm^3^).

## Results and discussion

### General considerations

Our aim in this study was to develop a compartmentalised in vitro model of the GI tract to investigate the bioavailability of orally consumed compounds. Static cell culture systems have generally been applied for absorption studies, which may not always be optimal for predicting in vivo absorption behaviour^10,32,33^. These systems also don’t allow for the inclusion of hands-off digestive sample processing. In our case, the digestive compartment is based on a well-established batch-based in vitro digestion approach, using fermenters having volumes of 10–100 mL. The use of batch-based systems implies the intermittent sequential addition of artificial digestive juices to mimic the different environments that an ingested sample finds itself in in the GI tract. Transfer of digested material to the absorption model cannot be performed automatically. We have chosen to implement this digestive approach in a flow-through system to facilitate automation and impart the freedom to dynamically change conditions in the different stages as desired to achieve enhanced versatility of the in vitro model^12^. Our system is unique by virtue of the fact that it combines microfluidic digestion-on-a-chip, an in vitro intestinal epithelial barrier model and online MS analysis in one automated total analysis system. Besides automation, the application of flow to transport and process sample from the mouth through to the intestine and MS analysis also means that the epithelial cell culture in the absorption module is nourished and maintained under more in vivo-like conditions.

We faced three challenges in the construction and demonstration of our system, namely: (1) the coupling of compartments having different internal volumes and thus operated at different flow rates, (2) the need for automated sample clean-up for MS, and (3) cell damage in the absorption module resulting from exposure to chyme. Above all, maintaining a relevant biological barrier is essential for studying the uptake of compounds. How we addressed these three challenges to ensure sequential in vitro digestion and absorption is described in the next three sections.

### Assembly of the total analysis system: resolving flow rate incompatibilities

In Fig. 1, a schematic representation is given of our three hyphenated compartments. Figure 1a depicts Compartment 1, the microfluidic digestion-on-a-chip system, consisting of three ‘chaotic’ micromixers representing the three phases of digestion in the mouth, stomach and intestine. In the first micromixer, the sample containing the compound of interest (1 µL/min) was mixed with artificial saliva (4 µL/min), resulting in an effluent flow of 5 µL/min from the mouth phase. The mouth phase was connected to the stomach phase by PTFE tubing, creating an incubation time in the oral phase of 2 min dictated by the internal volume of the tubing. In the second micromixer, artificial gastric juice (8 µL/min) was mixed with the 5 µL/min effluent from the mouth, with a gastric incubation time of 120 min determined by the volume of the tubing used to connect the stomach micromixer with the intestinal micromixer. Finally, the 13 µL/min effluent from the gastric phase was mixed with the intestinal juices (12 µL/min) in the third micromixer, resulting in a final chyme flow rate of 25 µL/min at the outlet of the digestion-on-a-chip. The incubation time of chyme in the intestinal phase was also 120 min, again dictated by the volume of the PTFE tubing connecting the intestinal phase with the cell culture barrier compartment. All microfluidic chips were kept at a constant temperature of 37 °C for optimal enzymatic activity. The selected flow rates and residence times in the different microreactors were based on average values that are relevant for in vivo human physiology^34–36^. Also, the ratios between the respective flow rates represent the in vivo volumetric ratios of the digestive phases.

The internal volume of the absorption module is 4 mL, 2 mL for the apical chamber and 2 mL for the basolateral chamber. The output flow rate of the digestion-on-a-chip therefore needs to be supplemented substantially in order to provide a sufficiently high flow rate to operate the absorption module at a physiologically relevant shear stress for the cells. The chyme from the digestion-on-a-chip (25 µL/min) was therefore mixed with transport buffer HBSS (175 µL/min) using a fourth micromixer, resulting in an eight-fold dilution of the chyme on the apical side of the cells. Note that at this point in the sample processing, the original sample solution had been diluted 200 times in total (25 times during passage through the digestion-on-a-chip, 8 times in the fourth micromixer). An important consideration for the samples analysed is thus that the compounds of interest are sufficiently soluble in the sample solution to achieve reasonably high initial concentrations to be detectable after processing-related dilution. Related to this, the detection limit of the final analysis method must be sufficiently low. The effluent from the fourth micromixer was connected to the apical side of the flow-through transwell (Fig. 1b) with a total flow rate of 200 µL/min, causing a realistic shear stress on the cultured epithelial cells in accordance with the in vivo range for the intestine (0.002–0.08 dyne/cm^2^) ^18,29^. Finally, the effluent from the flow-through transwell was connected either to a fraction collector or to the automated online analysis system (Compartment 3, Fig. 1c).

### Automated sample clean-up

In our previous work, a flow-through transwell barrier model was also coupled to MS detection, and sample dissolved in HBSS matrix was added to the apical chamber of the absorption module^23^. In this study, however, we have subjected our sample to in vitro digestion first, using the digestion compartment presented earlier^12^. The sample thus finds itself in a chyme matrix, which includes not only physiological concentrations of nutrients and ions, but enzymes and bile salts as well. MS analysis of chyme will result in increased chemical interference compared to previous studies where the sample was dissolved in cleaner HBSS buffer. This is an important consideration if UPLC-QTOF-MS is to be used as an online detector.

As mentioned above, the chyme was diluted by a factor of 8 with HBSS buffer before entering the absorption module. Besides ensuring physiological shear rates for the cell culture, this dilution also served to lower concentrations of species in the chyme matrix that cause higher background signal in the MS analysis. After the absorption module, the effluent flows were passed through C8 trap columns to accumulate compounds of interest and wash away interfering proteins. The compounds of interest were then analysed as described in our previous study using a set-up described in the Materials and Methods section and shown in Fig. 1c. Despite dilution, a higher background caused by the increased complexity of the sample matrix was observed. However, it was still possible to record mass spectra and reconstructed ion chromatograms of characteristic drug ions.

### In vitro intestinal barrier integrity upon exposure to chyme

The cell media used conventionally for absorption studies do not contain digestive compounds (enzymes and bile salts). However, because the absorption model in our system is preceded by a digestion compartment that produces chyme, it was crucial to investigate the effects of chyme on the barrier integrity of the cell layer, both before and after the experiments. Pure chyme is toxic to the Caco-2 and HT29-MTX-E12 cells used in our flow-through transwell model^24,37^. We therefore undertook a study in which this co-culture was exposed to differing dilutions of chyme in order to determine a non-toxic chyme composition. This investigation used three different techniques.

**Figure 2:**
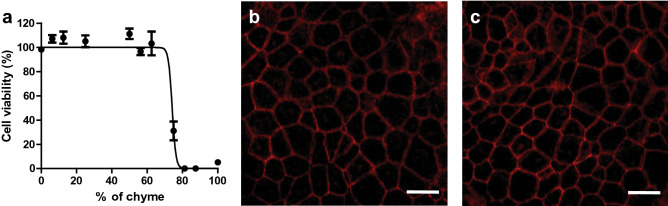
(**a**) Cell viability of Caco-2/HT29-MTX-E12 co-culture after 24 h exposure to increasing concentrations of chyme, measured using the WST-1 mitochondrial activity assay. Viability is given as a percentage of the control (% ± standard error of the mean (SEM); triplicates). A nonlinear curve was fitted through the points for clarity using GraphPad Prism. (**b**) Confocal image of Caco-2/HT29-MTX-E12 cells cultured in a transwell for 21 days (control). (**c**) Confocal image of Caco-2/HT29-MTX-E12 cells cultured in a transwell for 21 days and exposed to 12.5% chyme for 24 h. All exposures under static conditions. Cells were stained for tight junction protein ZO-1/TJP1 (red). Scale bar: 20 µm.

First, to determine the toxicity of the mixture of digestive juices coming from the digestion-on-a-chip, we used a WST-1 viability assay on proliferating cells to assess the mitochondrial activity as a measure of cell viability. Intestinal cell cultures were exposed to varying concentrations of chyme for 24 h. As shown in Fig. 2a, cell viability remained unaffected after 24 h exposure for chyme concentrations up to 62.5% (v/v) in medium. These results are comparable with a previous in vitro study^37^, suggesting that the living cells in Compartment 2 can be exposed to a mixture containing chyme.

For experiments with the digestion-on-a-chip connected to the flow-through transwell, pure chyme was diluted by a factor of 8 in HBSS to achieve the desired flow rate (200 μL/min) for the apical compartment. This yielded a 12.5% chyme solution in HBSS, which is well below the upper acceptable concentration of 62.5% reported above to maintain proper cell viability during experiments. Our choice of a chyme concentration below 62.5% is supported by data from Mahler et al.^24^ using a microfluidic gut-on-a-chip system. Their study used a chyme solution, originally described by Glahn et al.^38^, that exhibited an enzymatic activity that was equivalent to 77% of the enzymatic activity of our chyme (based on pancreatin content in the solution that is presented to the cells). They observed cell damage (indicated by a drop in both viability and trans-epithelial electrical resistance) after exposing a Caco-2/HT-29MTX co-culture to the chyme solution for only 2 h. In some cases, damage was observed after even shorter exposure times. In the in vivo situation, uptake of compounds may start at the same time as intestinal digestion (i.e., upon entering the duodenum), especially in fasted state and less pronounced in the fed state of our model. However, undiluted chyme is toxic to the cells in our absorption compartment, so it must be diluted before entering that compartment. Dilution of chyme causes the concentration of enzymes contained in the chyme to decrease, with an associated decrease in enzyme activity, i.e. reduced digestive capacity. This is why digestion and absorption cannot be performed concurrently in our model. This is in agreement with in vitro digestive systems that are currently used to digest samples before doing uptake studies^35,36^.

For our second series of experiments, we statically exposed a 21-day old co-culture of Caco-2 and HT29-MTX-E12 cells to 12.5% chyme for 24 h and subsequently stained the tight junction protein ZO-1/TJP1. Co-cultured cells were exposed to 0% chyme (control, Fig. 2b) and to 12.5% chyme (Fig. 2c), and an interconnecting network of tight junction proteins is shown in red. No differences in the quality of the tight junctions were observed for the two co-cultures, indicating good barrier integrity. Moreover, cell barrier integrity was also confirmed after each permeability experiment using the fluorescent marker, lucifer yellow, which is not transported by the cells. Any translocation of this compound to the basolateral compartment amounting to more than 5% of the total amount present in the apical compartment thus indicates leakiness of the barrier. Cell layers were exposed to 500 µg/mL lucifer yellow in HBSS for 30 min after every permeability experiment. Cell layers allowing more than 5% of lucifer yellow to translocate to the basolateral side were considered leaky, and data from these cultures were discarded. The cell barriers used for calculating permeability showed 0.9 ± 0.4% lucifer yellow transport, confirming that the biointegrity of living cells can be fully maintained in the presence of chyme.

### In vitro digestion and intestinal permeability of omeprazole

**Figure 3:**
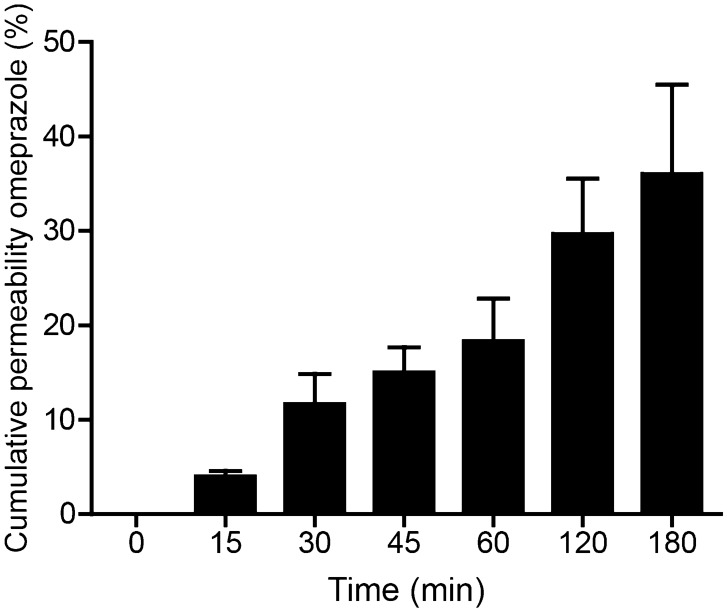
Permeability of omeprazole across a monolayer of Caco-2/HT29-MTX-E12 cells in a static transwell, without digestive juices in the apical matrix. Permeability is given as a percentage of the initial apical concentration (% ± SEM; n = 3).

To evaluate digestion-on-a-chip in combination with our dynamic model of the intestinal barrier, we used the model drug compound, omeprazole (molecular structure, Fig. S1, SI). Omeprazole is a proton pump inhibitor that irreversibly blocks the last step of acid production in the stomach wall, thereby increasing the gastric pH^39^. Omeprazole is preferably administrated orally via a suspension, tablet or capsule. As omeprazole itself is acid-labile, these drug formulations contain an enteric coating to protect omeprazole from acid degradation in the stomach^27^. Omeprazole is then released in the small intestine and absorbed. Prior to evaluation of the combined set-up comprising digestion and cellular uptake of omeprazole, we determined a 5 µg/mL concentration of this drug to be non-toxic to the cell co-culture, using the WST-1 assay (Fig. S2, SI). Static co-cultures of Caco-2 and HT29-MTX-E12 cells were then exposed to omeprazole at 5 µg/mL for 3 h. In Fig. 3, the cumulative percentage of omeprazole that has crossed the cell barrier to the basolateral side is given at different time points, reaching 36.1% of the apical concentration after 3 h. The apparent permeability coefficient (P_app_) was calculated to be 54.9 ± 12.9 × 10^–6^ cm/s, which is in the same range as in vitro P_app_ data found for omeprazole in the literature (13.4–53.2 × 10^–6^ cm/s, for monolayers of Caco-2 and L-MDR1 cells)^40,41^. Extrapolation of data obtained in vitro to the in vivo situation remains difficult^42^, but the Biopharmaceutics Classification System (BCS)^43^ that is used by North American and European authorities discriminates drugs based on their in vitro permeability with respect to the drug, metoprolol, of which the P_app_ is in the order of 30–50 × 10^–6^ cm/s^44^. Both the in vivo and in vitro permeability of drugs in the BCS have been evaluated extensively, and the correlation between in vitro and in vivo permeabilities has been established. In other words, if the in vitro permeability of a drug is higher than that of metoprolol, the drug is considered highly permeable in vitro and likely is highly permeable in vivo as well. This is the case for omeprazole, which we too find to have a high in vitro permeability. A next step in the extrapolation of data obtained from in vitro models as described in this work to the in vivo situation would be their use in so-called Physiologically Based Kinetic Models (PKB models) and Quantitative in vitro to in vivo Extrapolations (QIVIVE)^45,46^. These models use in vitro intestinal uptake rates as input in addition to other human physiological parameters, and have been extensively exploited to support safety assessments of chemicals.

**Figure 4:**
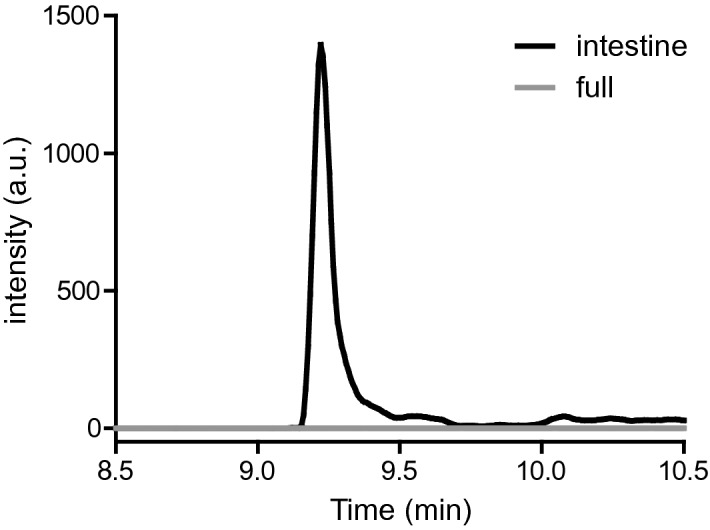
Reconstructed-ion chromatogram of *m/z* 346 ([M + H]^+^) at time point 90 min on the apical side after only intestinal digestion (black) (Fig. 1b) or full digestion (grey) (Fig. 1a,b). Samples were collected by a fraction collector followed by offline analysis using chip-based UPLC-QTOF-MS.

Next, we coupled digestion-on-a-chip to the dynamic model of the intestine, using two different set-ups. In the first set-up, all three chaotic micromixers simulating the mouth, stomach and intestine were implemented, and connected via a fourth micromixer to the flow-through transwell, using the compartmental set-up depicted in Fig. 1a,b. Every minute, apical and basolateral samples were collected in two separate 96-well plates using two fraction collectors. In the second set-up, we emulated the working mechanism of an enteric-coated tablet of omeprazole, which only releases omeprazole in the intestinal compartment to prevent exposure to gastric acid. This was done by excluding the mixers for the mouth and stomach compartments to realise a simplified version of chip-based digestion. Only one micromixer was used to mix the sample (omeprazole) and the pre-mixed digestive juices (saliva, gastric juice, and intestinal juice). After dilution in the fourth mixer and perfusion of the dynamic cell coculture, samples were collected from the apical and basolateral side in two separate 96-well plates. In Fig. 4, the reconstructed ion currents of the [M + H]^+^ ion of omeprazole at *m/z* 346 are given for both complete digestion and exposure to only intestinal digestion after 90 min. The figure clearly shows that the unprotected omeprazole is fully degraded in the total digestion system, in accordance with expectations; no signal remains for the *m/z* 346 ion (in grey). We did not observe any clear degradation products of omeprazole in the MS data^47^.

In the second experiment mimicking the ingestion of enteric-coated omeprazole, we clearly observed the omeprazole ion in the apical effluent (Fig. 4, in black), as expected. However, we did not observe any translocation of omeprazole to the basolateral site. This is in contrast to the static permeability data for omeprazole (Fig. 3), and in vivo data which predict that uptake of omeprazole could be expected in the dynamic flow-through system^40,41^. From the literature, it is known that omeprazole heavily binds to plasma proteins^48^. A control experiment was conducted to examine the effect of digestive juices (chyme) on the translocation of omeprazole in a static transwell (Fig. S3, SI). It was found that the uptake of omeprazole in the presence of digestive juices was about three times lower compared to omeprazole dissolved in only HBSS buffer. This may explain why no omeprazole was detected in the basolateral compartment of the flow-through transwell after fraction collection and offline analysis. Translocation appears to have been lowered due to binding with proteins in the chyme matrix. Moreover, omeprazole will generally be more difficult to detect in the flow-through case than in the static case, as in a dynamic system no accumulation of the translocated compound occurs due to the collection of samples every minute.

### Compartmentalised in vitro GI tract with online analysis: proof-of-principle with and without co-exposure to a food matrix

**Figure 5:**
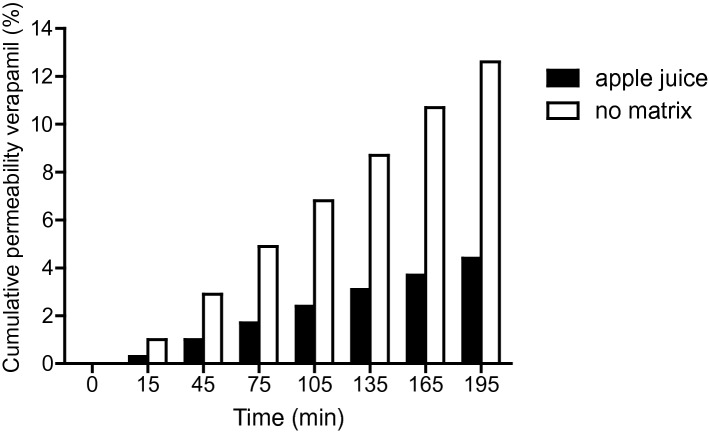
Permeability of verapamil in apple juice matrix (black) or no food matrix (ultra-pure water, white) across a monolayer of Caco-2/HT29-MTX-E12 cells, measured in the integrated GI tract with online analysis set-up combining the three compartments in Fig. 1a–c. Permeability is given as a calculated cumulative percentage of the starting apical concentration (% ± SEM; n = 1).

All the compartments of our system (digestion-on-a-chip, flow-through transwell with co-cultured intestinal cells, and MS analysis) were combined in one hyphenated, online system (Fig. 1a–c), creating a multi-module GI tract with automated online analysis to monitor in vitro oral bioavailability over time. In a previous study^23^, the flow-through transwell was combined with online MS analysis. In this study, we further challenged the system by including microfluidic digestion-on-a-chip (Fig. 1a), allowing pre-treatment of samples with digestive juices before studying translocation through a model of the gut wall. We used the model compound, verapamil (molecular structure, Fig. S4, SI), a drug for treatment of high blood pressure and other conditions, for evaluation of the modular in vitro GI tract. Verapamil has the advantage that there are plenty of data available in the literature for both static and dynamic transwell systems, making it possible to benchmark our system^23,49,50^. First, we examined if verapamil is affected by digestion in the different phases by performing a test tube digestion. As can be seen in Fig. S5 in the supplementary information, verapamil is not affected by digestion. Therefore, it was hypothesised that verapamil would exhibit similar behaviour in our modular in vitro GI tract compared to its behaviour in the earlier flow-through set-up reported previously^23^. Figure 5 shows the cumulative permeability of verapamil over 195 min measured in the entire system shown in Fig. 1a–c (in white). The results are very similar to the data from Santbergen et al. indicating that including the additional digestion-on-a-chip functionality affects neither the biointegrity of the co-culture of Caco-2 and HT29-MTX-E12 cell model, nor the overall analytical performance^23^. In contrast to the reduced absorption of omeprazole in the presence of digestive juices, the translocation of verapamil is not affected at all.

To emulate the functions of the GI tract even further, a final experiment was performed in which verapamil was not administered in ultrapure water, but in apple juice as a simplified food matrix. Apple juice was chosen because it is a reasonably complex, liquid food matrix suitable for the setup (i.e., liquid form), containing sugars and minerals in water. The absence of mastication in the oral phase of our system does not allow for samples based on solid foods, and special care must be taken in order to keep the microchannels free of blockages by precipitates. In Fig. 5, the uptake of verapamil dissolved in apple juice (black) is depicted versus the control in water (white). Clearly, the absorption of verapamil is much slower in the presence of an apple juice matrix compared to the control. It is well known that food (or certain food ingredients) alters the bioavailability of drugs, for example by drugs binding to proteins, fats, or calcium ions contained in food^51,52^. Fruit juices have been shown to inhibit the transport of drugs into cells by organic anion-transporting polypeptides (OATPs) in cell membranes^53^. However, since verapamil is mainly transported via passive diffusion^54^, this effect was not expected to play a role in this study. Nevertheless, the uptake of verapamil seems to be affected by apple juice in this proof-of-principle experiment, and more experiments with different sample matrices are required to ascertain if OATPs are involved in verapamil transport after all, or if there is another as yet unidentified effect taking place.

## Conclusion

The integrated compartmentalised model of the GI tract described in this paper comprises pretreatment of samples with digestive juices, followed by absorption of sample molecules and their possible metabolites through an in vitro intestinal epithelial barrier. Online coupling to UPLC-QTOF-MS resulted in an automated online read-out of oral bioavailability parameters of the molecules. To the best of our knowledge, this system is the first of its kind developed for assessment of oral bioavailability parameters in pharmacological and toxicological applications. This system encompasses the two key processes of the human intestinal tract, namely digestion and absorption. The sequential combination of different compartments having different volumes has been demonstrated, by adjustment of volumetric flow rates between compartments. The physiological relevance of this system has been improved by the use of medium which has been made to resemble dilute chyme. By applying an automated sample clean-up system developed in a previous study^23^, it was possible to analyse the compounds of interest in dilute chyme using MS detection.

There are few previous studies which have pursued this total-analysis-system route to better mimic the in vivo situation^24,25,37^. While development of such a system requires some effort, a good system may lead to a significant reduction in the need for animal models in these types of studies. This is certainly relevant now, at a time where many companies are committing to doing far fewer animal experiments. For future applications, the incorporation of cell types that have higher metabolic capacity than Caco-2 cells is desired. In particular, 3-D culture formats such as organoids could greatly improve the applicability of the system^55^. In silico models are being developed, but they rely heavily on data that have been obtained in vivo or in in vitro systems that extrapolate to the in vivo situation. Systems like ours will be required to optimise these in silico models^42,56,57^.

In this work, we show maximum versatility for our in vitro model. The complete system as described can be used to study many physiological processes that involve digestion and absorption of nutritionally as well as pharmacologically important compounds. There is one caveat associated with the system, namely that it is unsuited for mimicking in vivo processes in which the digestion of a compound in the intestine actually drives its absorption through the creation of high local concentrations. (One such process is the supersaturation of lipid-based drug delivery systems in vivo, the investigation of which would require a different model in vitro^58^.) Each compartment can be tailored to specific applications according to the needs of end-users, including residence times and digestive juice compositions. An example of this in this paper is the simple customisation of the digestive system for the study of omeprazole. Another route for future research is to include drugs that are wholly or partially affected by enzymatic action, for example in the conversion of prodrugs into active drugs. Finally, the flow-through nature of our hyphenated system has potential for the automation of oral bioavailability testing in drug development (novel drug and drug formulation development, next generation risk assessment) and chemical toxicity testing.

## Supplementary Information

Supplementary Information
