# Supplementary material for: A versatile, compartmentalised gut-on-a-chip system for pharmacological and toxicological analyses
Source: Sci Rep. 2021 Mar 1;11:4920. doi: 10.1038/s41598-021-84187-9 (PMC7921645; doi:10.1038/s41598-021-84187-9)
Supplement: Supplementary file 1 — Supplementary Information [file 41598_2021_84187_MOESM1_ESM.pdf]

Supplementary Information

A versatile, compartmentalised gut-on-a-chip system for pharmacological and toxicological analyses

Pim de Haan [a,b,‡], Milou J.C. Santbergen [b,c,‡], Meike van der Zande [d], Hans Bouwmeester [e], Michel W.F. Nielen [c,d] and Elisabeth Verpoorte [a*]

[a] University of Groningen, Groningen Research Institute of Pharmacy, Pharmaceutical Analysis, P.O. Box 196, XB20, 9700 AD Groningen, The Netherlands.

[b] TI-COAST, Science Park 904, 1098 XH Amsterdam, The Netherlands

[c] Laboratory of Organic Chemistry, Wageningen University, Stippeneng 4, 6708 WE Wageningen, The Netherlands

[d] Wageningen Food Safety Research, Wageningen Research, P.O. Box 230, 6700 AE Wageningen, The Netherlands

[e] Division of Toxicology, Wageningen University, Stippeneng 4, 6708 WE Wageningen, The Netherlands

* Corresponding author: E-mail: e.m.j.verpoorte@rug.nl

[‡]These authors contributed equally to this work.

Table of Contents

Supplemental Methods 3

Supplemental Figures 4

Supplemental Methods

Artificial digestive juices

Table S1 Optimized composition of the artificial digestive juices, dissolved in ultrapure water (De Haan et al.[1])

**Compound**	**Saliva (mg/L)**	**Gastric Juice (mg/L)**	**Duodenal juice (mg/L)**	**Bile (mg/L)**
$CaCl_2$		302	151	167.5
Glucosamine HCl		330		
Glucose		650		
Glucuronic acid		20		
KCl	896	824	564	376
KH_2PO_4			80	
KSCN	200			
$MgCl_2.6H_2O$			50	
Na_2SO_4	570			
NaCl	298	2752	7012	5259
$NaH_2PO_4.H_2O$	1021	306		
$NaHCO_3$			3388	5785
NH_4Cl		306		
Urea	200	85	100	250
Uric acid	15			
HCl		4.16 mM	5.57 mM	6.17 mM
NaOH	2.9 mM			
α-Amylase (*Bacillus sp.*)	145			
Bile (bovine)				6000
Lipase (porcine pancreas)			500	
Pancreatin (porcine pancreas)			3000	
Pepsin (porcine gastric mucosa)		1000		

[1] P. de Haan, M. A. Ianovska, K. Mathwig, G. A. A. van Lieshout, V. Triantis, H. Bouwmeester and E. Verpoorte, *Lab Chip*, 2019, **19**, 1599-1609

Supplemental Figures

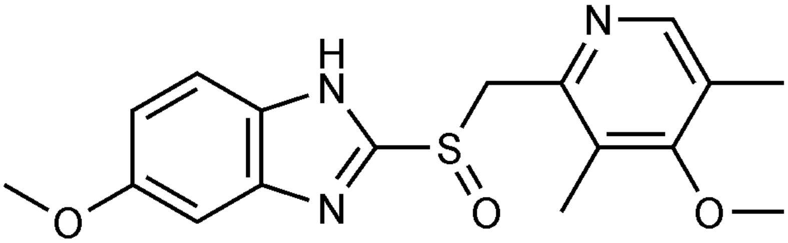

Figure S1: Molecular structure of omeprazole.

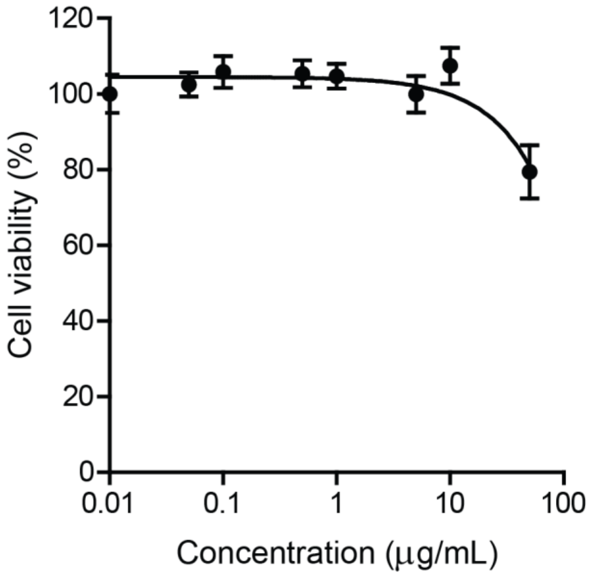

Figure S2: Cell viability of a Caco-2/HT29-MTX-E12 co-culture after 24 h exposure to increasing concentrations of omeprazole using the WST-1 mitochondrial activity assay. Viability is given as a percentage of the control (% ± SEM; n=3).

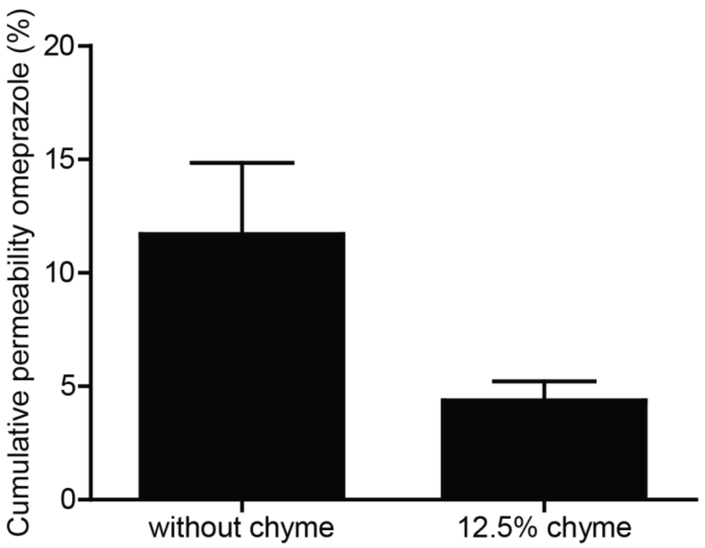

Figure S3: Permeability of omeprazole in the absence and presence of 12.5% chyme (composition as mentioned in table S1) across a monolayer of Caco-2/HT29-MTX-E12 cells in a static transwell after 30 minutes. Permeability is given as a percentage of the apical concentration (% ± SEM; triplicates).

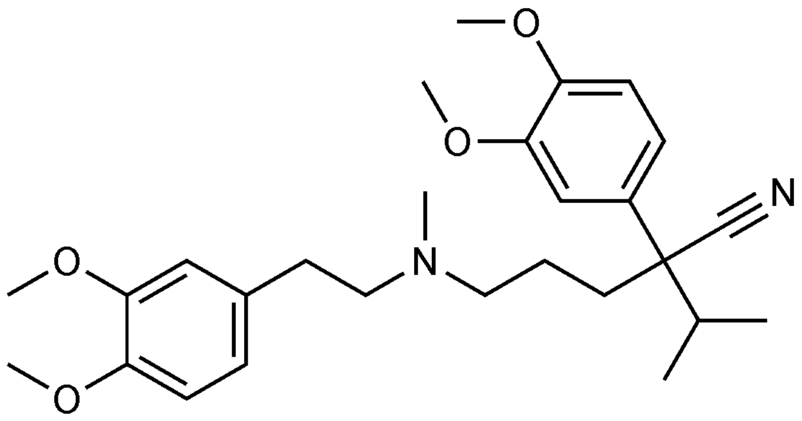

Figure S4: Molecular structure of verapamil.

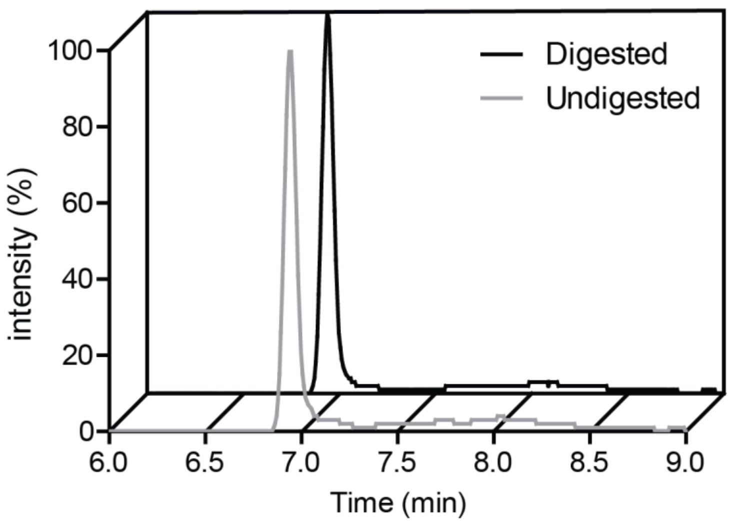

Figure S5: Reconstructed-ion chromatogram of *m/z* 455 $[M+H]^+$ ion of verapamil after digestion (conditions as mentioned in table S1) in a test tube (black) or no digestion (grey).
